# Hyaluronan Signaling during Ozone-Induced Lung Injury Requires TLR4, MyD88, and TIRAP

**DOI:** 10.1371/journal.pone.0027137

**Published:** 2011-11-04

**Authors:** Zhuowei Li, Erin N. Potts-Kant, Stavros Garantziotis, W. Michael Foster, John W. Hollingsworth

**Affiliations:** 1 Department of Medicine, Duke University, Durham, North Carolina, United States of America; 2 Department of Immunology, Duke University, Durham, North Carolina, United States of America; 3 National Institute of Environmental Health Sciences, Research Triangle Park, North Carolina, United States of America; National Jewish Health, United States of America

## Abstract

Ozone exposure is associated with exacerbation of reactive airways disease. We have previously reported that the damage-associated molecular pattern, hyaluronan, is required for the complete biological response to ambient ozone and that hyaluronan fragments signal through toll-like receptor 4 (TLR4). In this study, we further investigated the role of TLR4 adaptors in ozone–induced airway hyperresponsiveness (AHR) and the direct response to hyaluronan fragments (HA). Using a murine model of AHR, C57BL/6J, TLR4−/−, MyD88−/−, and TIRAP−/− mice were characterized for AHR after exposure to either ozone (1 ppm×3 h) or HA fragments. Animals were characterized for AHR with methacholine challenge, cellular inflammation, lung injury, and production of pro-inflammatory cytokines. Ozone-exposed C57BL/6J mice developed cellular inflammation, lung injury, pro-inflammatory cytokines, and AHR, while mice deficient in TLR4, MyD88 or TIRAP demonstrated both reduced AHR and reduced levels of pro-inflammatory cytokines including TNFα, IL-1β, MCP-1, IL-6 and KC. The level of hyaluronan was increased after inhalation of ozone in each strain of mice. Direct challenge of mice to hyaluronan resulted in AHR in C57BL/6J mice, but not in TLR4−/−, MyD88−/−, or TIRAP−/− mice. HA-induced cytokine production in wild-type mice was significantly reduced in TLR4−/−, MyD88−/−, or TIRAP−/− mice. In conclusion, our findings support that ozone-induced airway hyperresponsiveness is dependent on the HA-TLR4-MyD88-TIRAP signaling pathway.

## Introduction

Inhaled ozone significantly contributes to both human morbidity and mortality and is estimated to annually account for approximately 800 premature deaths, 4500 hospital admission, 900000 school absences and more than a million restricted activity days with an estimated $5 billion annual economic burden [1]. There does not appear to be a lower threshold level of ozone associated with detrimental health effects [2], [3], [4] with each 10 ppb increase in daily ozone being associated with approximately 0.87% increase in total mortality [5]. It is anticipated that levels of ambient ozone will rise with predicted changes in global climate [6]. Understanding the health impacts of ambient ozone is therefore of high clinical interest. Furthermore, understanding the molecular mechanisms that regulate the biological response to inhaled ozone can provide insight into oxidant-related lung injury and reactive airways disease, which could identify novel therapeutic approaches to asthma.

Toll-like receptors (TLRs) are membrane-spanning receptors that recognize structurally conserved molecules that are usually derived from microbes. However, diverse environmental exposures by an inhalational route are also known to activate innate immunity through TLRs, and can thus exacerbate existing allergic airways disease [7], [8], [9] and contribute to airway hyperresponsiveness [10], [11]. For example, toll-like receptor 4 (TLR4) is required for the complete biological and functional responses of airway and lung tissues to inhaled ozone. TLR4, as a candidate gene in response to ozone, was initially identified through forward genetics and supported by the C3H/HeJ (TLR4-mutant) animal, which is protected from sub-chronic exposure to ozone-induced lung injury [12], [13]. TLR4-deficient animals were subsequently identified to be protected from ozone-induced airway hyperresponsiveness [11]. TLR4 activates major signal transduction pathways through distinct adaptor proteins capable of contributing to cellular responses specifically myeloid differentiation primary response protein 88 (MyD88) and TIR domain-containing adapter protein for TLR4 (TIRAP). Previous work suggests that MyD88 is required for the complete response to ozone [14] and sub-chronic exposure to ozone results in increased mRNA expression of MyD88 [15]. However, the biologic mechanisms whereby these genes of innate immunity contributed to the response to oxidant lung injury remained elusive.

Previous work supports that the extracellular matrix component, hyaluronan, can function as a signaling molecule when fragmented into low molecular weight [16]. Additionally, hyaluronan can function as an endogenous ligand to TLR4 in context of sterile (non-infectious) tissue injury [17]. Oxidant stress is one mechanism that results in fragmentation of hyaluronan [18], [19]. We have recently identified that hyaluronan fragments contribute to the complete biological response to ozone [20]. Furthermore, fragments of hyaluronan function as an endogenous ligand of TLR4 in context of several forms of oxidant lung injury including bleomycin [21], asbestos [22], and ozone [23]. However, the intracellular adaptor molecules required to facilitate hyaluronan-dependent cell signaling remain unknown.

We hypothesized that ozone inhalation results in generation of immunostimmulatory fragments of hyaluronan that signal in a manner dependent on TLR4 and the cytoplasmic adaptor proteins, MyD88 and TIRAP. Similar to prior reports, we identify that both TLR4 and MyD88 are required for development of ozone-induced AHR. We now identify that the adaptor molecule TIRAP is additionally required for development of ozone-induced AHR. The profile of pro-inflammatory cytokines after inhalation of ozone is similar in TLR4−/−, MyD88−/−, and TIRAP−/− mice. Direct challenge of mice to fragments of hyaluronan identifies a role of both MyD88 and TIRAP in development of AHR and release of pro-inflammatory cytokines. These studies provide novel insight into the intracellular adaptor molecules required for the development of airway hyperresponsiveness in response to the endogenous TLR4-ligand hyaluronan.

## Materials and Methods

### Animal

All experimental protocols were reviewed and approved by the Institutional Animal Care and Use Committee at Duke University Medical Center and performed in accordance with the standards established by the U.S. Animal Welfare Acts. Animals were treated humanely and with regard for alleviation of suffering. Animal work is covered under the following registrations, permits, and accreditations: United States Department of Agriculture (Registrant 55-R-0003 #83); The National Institutes of Health (Assurance 3195-01); and the Association for the Assessment and Accreditation of Laboratory Animal Care, International (File #363). 6–8 weeks old male C57BL/6J mice were purchased from Jackson Laboratory (Bar Harbor, ME). TLR4−/−, MyD88−/−, and TIRAP−/− mice were generously provided by S. Akira [24], [25], [26] and then backcrossed onto C57BL/6J for at least ten generations. Presented results are representative of at least two experimental repeats utilizing age and gender matched animals.

### Ozone Exposure

C57BL/6J, TLR4−/−, MyD88−/− and TIRAP−/− mice were exposed to 1 part per million (ppm) of ozone for 3 h as previously described [27] and phenotyped 20–24 hours after initiation of exposure. The dose and exposure duration were selected based on similar biological response observed in human exposure studies, published deposition fraction data for O_3_ [28], [29], and our previous observations in the mouse model [27]. Under this exposure dose, wild-type mice maintained comparable airway responsiveness to ozone with less lung inflammation, when compared with higher doses of ozone used in previous publications [20], [23]. Briefly, animals in separate caging were placed into a 55-L Hinner-style chamber and exposed to filtered air (FA) or ozone. Ozone was generated by directing 100% oxygen through a UV light ozone generator and mixed with filtered air before supply. Environmental conditions during exposures included air temperatures between 20 to 22°C, relative humidity at 50 to 60% and approximately 20 exchanges of chamber air per hour. Chamber ozone concentration was continuously monitored with a UV light photometer.

### HA challenge

Endotoxin-free HA fragments were prepared as previously described [20]. Briefly, high molecular weight hyaluronan (Healon GV, AMO, Santa Ana, CA) was reconstituted to 0.5 mg/ml in 0.02 M acetate/0.15 M sodium chloride, pH 6.0. For the production of low molecular weight hyaluronan, Healon was sonicated 3 seconds for a total of 3 times on ice using a sonic dismembrator (Thermo Fisher Scientific Inc., Pittsburgh, PA). The sizes of HA were confirmed by agarose gel electrophoresis. For *in vivo* experiments, animals were briefly anesthetized by inhalation of isoflurane; 50 µl of 0.5 mg/ml of HA or same amount of vehicle control was then instilled into the lung by oropharyngeal aspiration. This protocol and dose of hyaluronan fragments are used to model ozone-induced reactive airways disease [20]. Animals were allowed to recover for two hours before measurement of AHR.

### Whole lung lavage, cell counts and analysis

As previously described [20], mice were euthanized with CO_2_ and the lungs were fully inflated three times serially to 25 cmH_2_O of 0.9% NaCl. All three lavage returns were pooled together for the following analysis. Cell counts were performed using a hemocytometer and differentials were performed by H&E stained cytospins. Cytokine/chemokines IL-1β, IL-6, KC, MCP-1 and TNFα were determined by Luminex (Bio-Rad, Hercules, CA) using 5-plex reagents from Millipore (Billerica, MA). Assay sensitivities are 1.8 pg/ml for IL-6, 2.0 pg/ml for IL-1β, 1.4 pg/ml for KC, 5.3 pg/ml for MCP-1, 1.0 pg/ml for TNFα. Total protein concentrations in lung lavage fluid were measured by the Lowry Assay (Bio-Rad, Hercules, CA).

#### Airway Physiology

Airway hyperresponsiveness to methacholine (Mch) challenge was measured 20–24 hours post ozone or air exposure. At this time point, acute ozone inhalation induced significant change in AHR to Mch challenge, which is similar to human response to ozone at 24 hours post exposure [30]. Before measurement, animal was anesthetized with 60 mg/kg of pentobarbital sodium by intraperitoneal injection. Mice were then given neuromuscular blockade (0.8 ml/kg pancuronium bromide) and ventilated with a computer-controlled small animal ventilator (FlexiVent; SCIREQ, Montreal, QC), with a tidal volume of 7.5 ml/kg and a positive end-expiratory pressure of 3 cm H_2_O. Lung total resistance was assessed using low frequency oscillation technique whereby airway pressure (via a tracheal cannula) and tidal volume data were generated by the application of a 2 second sine wave volume perturbation (amplitude ∼0.2 ml and frequency = 2.5 Hz) [31], [32]. Following baseline resistance measurements, mice were challenged in dose related manner with methacholine aerosol (Mch 10–100 mg/mL) and after each Mch dose the lung was hyper-inflated to return resistance to baseline levels. After baseline recruitment maneuvers to achieve total lung capacity, baseline total respiratory system resistance (R measurements) were collected and analyzed using the linear single-compartment model with multiple linear regression as described by the manufacturer (SciREQ, Montreal, Canada). The resistance measurements were then averaged at each dose and graphed (R_T_, measured in cmH_2_O/ml/s) along with the initial baseline measurement.

### Statistics

All data are expressed as mean ± SEM. Two–way ANOVA for comparisons among multiple groups was performed using Graphpad Prism 5.0. Student-t test was used for individual comparisons between groups. Significance was defined as two-tailed P value of less than 0.05. Presented results represent at least two replicate studies.

## Results

### Ozone-induced airway hyperresponsiveness is dependent on TLR4, MyD88 and TIRAP

**Figure 1:**
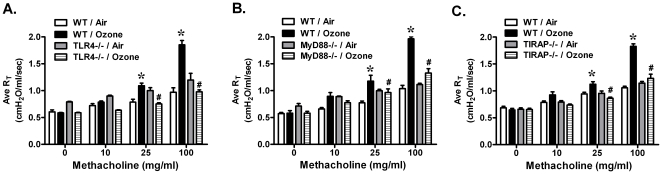
Ozone inhalation increased airway sensitivity to methacholine challenge and was dependent on the TLR4-MyD88-TIRAP signaling pathway. Animals were exposed to filtered air (FA) or 1 ppm of ozone for 3 hours. Airway responsiveness to methacholine challenge was measured 24 h later. **A**) Ozone-induced AHR was increased in WT mice but not in TLR4−/− mice, **B**) MyD88−/−mice, or **C**) TIRAP−/− mice (*p<0.05 vs. FA-WT; #p<0.05 vs. O3-WT, N = 5–6 per group).

TLR4, a critical cell surface receptor, mediates the increased airway reactivity to methacholine challenge in mice exposed to both 2 ppm and 3 ppm of ozone [11], [14], [23]. A lower dose of ozone, 1 ppm, was further reported by our group to be associated with increased AHR [27]. In this study, we investigated whether TLR4 is required for the response to 1 ppm ozone in the development of AHR and whether specific intracellular TLR4 adaptors are required for the response to ozone. After 20–24 hours recovery from inhalation of ozone at 1 ppm for 3 hours, we observed that ozone-exposed wild-type mice demonstrated significantly increased airway responsiveness to 25 and 100 mg/ml of methacholine challenge compared with filtered air exposed-mice. Ozone-exposed TLR4-deficient mice showed significantly reduced AHR when compared with ozone-exposed wild-type animals. Both air-exposed wild-type and TLR4-deficient mice have no difference in airway sensitivity to methacholine challenge (Figure 1A). Therefore, TLR4 contributes to AHR 24 hours after inhalation of 1 ppm ozone for 3 hours. We then investigated the role of TLR4 adaptor molecules in the pathogenesis of ozone-induced AHR. Using gene knockout mice, MyD88 has been reported to be involved in 3 ppm of ozone-induced AHR [14]. We demonstrated that MyD88 contributes to this lower dose ozone exposure (Figure 1B). We additionally investigated the contribution of TIRAP, which is another TLR4 adaptor protein previously reported to function through binding to MyD88 [33]. Similar to both the TLR4-deficient and MyD88-deficient animals, TIRAP-deficient mice failed to demonstrate enhanced AHR in response to methacholine challenge when compared with ozone-exposed wild-type mice (Figure 1C). There were no observed differences in methacholine sensitivity among air-exposed wild-type, TLR4−/−, MyD88−/− and TIRAP−/− animals (Figure 1B and 1C). AHR was also not different between ozone-exposed TLR4−/−, MyD88−/− and TIRAP−/− animals. Together these data support that TLR4, MyD88, and TIRAP contribute to ozone-induced AHR.

### TLR4, MyD88 and TIRAP partially contribute to ozone-induced inflammation

**Figure 2:**
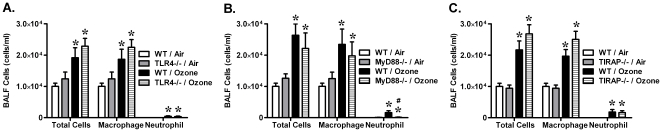
Ozone exposure increased neutrophilic lung inflammation in a manner partially dependent on MyD88, but not TLR4 and TIRAP. Ozone-exposed mice demonstrated increased neutrophil cell counts in BALF when compared to air-exposed animals. Neutrophil recruitment to the airspace was independent of TLR4 (**A**) and TIRAP (**C**), but was partially dependent of MyD88 (**B**) (* p<0.05, vs. FA exposed group; # p<0.05 vs. O_3_-WT, N = 4–6 per group).

**Figure 3:**
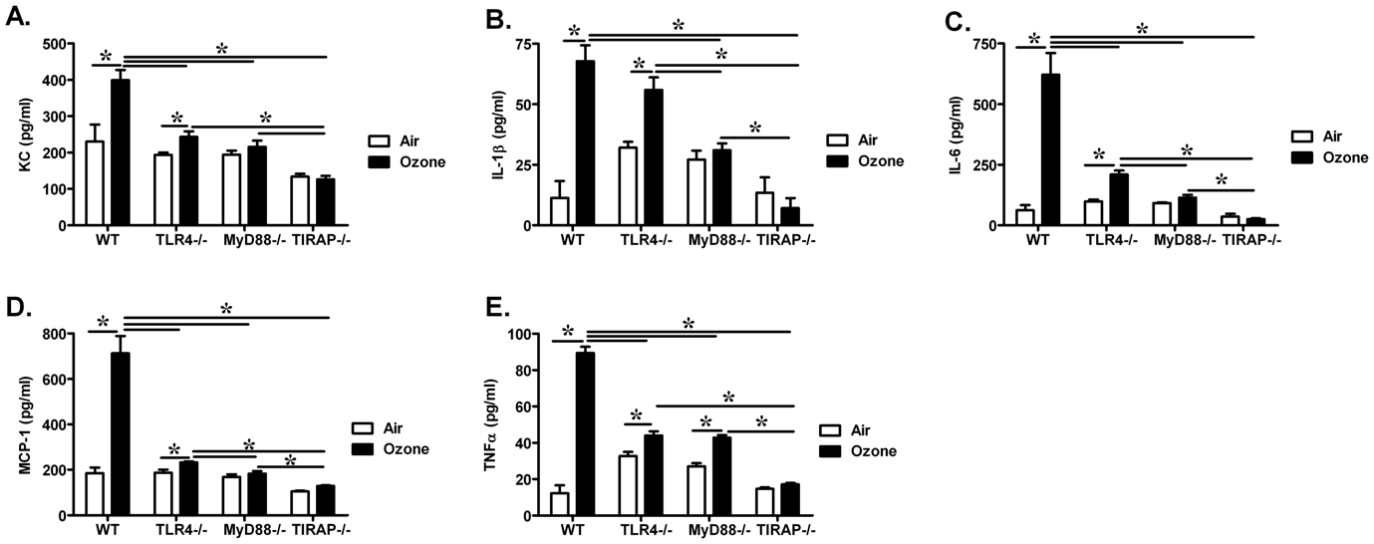
Ozone inhalation increased the level of pro-inflammatory factors in alveolar lavage fluid in a manner partially dependent on TLR4, MyD88, and TIRAP. The level of (**A**) KC, (**B**) IL-1β, (**C**) IL-6, (**D**) MCP-1 and (**E**) TNFα in BALF from air and ozone-exposed WT, TLR4−/−, MyD88−/− and TIRAP−/− mice were measured by luminex beads (*p<0.05, vs. FA exposed group; #p<0.05, vs. O_3_-exposed group comparisons between strains; N = 4–5 per group).

**Figure 4:**
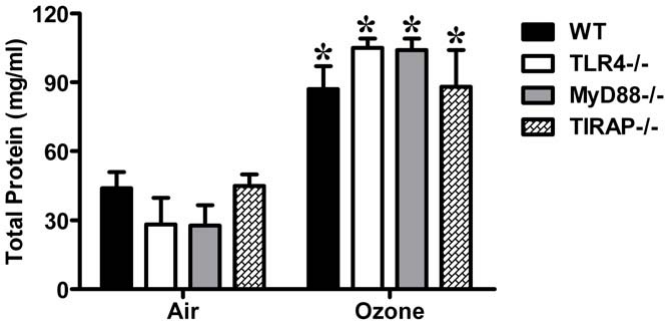
Increased lavage total protein level after ozone exposure was independent of TLR4, MyD88, and TIRAP (*p<0.01, vs. FA exposed group; N = 5–6 per group).

Inhalation of ozone is recognized to cause an increase in total cells, neutrophils, and pro-inflammatory cytokine/chemokine levels in BALF. Consistent with previous reports, 1 ppm of ozone exposure increased neutrophil infiltration into the alveolar compartments in wild-type mice. The number of neutrophils in the BALF was also increased in TLR4−/, MyD88−/−, and TIRAP−/− mice exposed to ozone when compared to air (Figure 2A, 2B and 2C). The number of neutrophils was reduced in MyD88−/− mice when compared to ozone-exposed wild-type mice (Figure 2B). This observation suggested that neutrophilic inflammation caused by ozone exposure was partially dependent on a MyD88-mediated pathway. However, we did not observe a role of either TLR4 or TIRAP in ozone-induced neutrophil recruitment into the airspace. We further measured the level of cytokine/chemokine levels in BALF (Figure 3A, 3B, 3C, 3D and 3E). The results demonstrated that the levels of KC, IL-1β, IL-6, MCP-1 and TNFα in BALF from ozone–exposed wild-type animals were each significantly higher than that in BALF from air-exposed control animals. By comparison, the levels of KC, IL-1β, IL-6, MCP-1 and TNFα were also increased in BALF from ozone-exposed TLR4−/− mice, when compared with air exposed controls, but significantly lower than levels in BALF from ozone-exposed wild-type mice. Elevated production of KC, IL-1β, IL-6 and MCP-1 was not detected in BALF from either MyD88−/− or TIRAP−/− mice after either ozone or air exposure. The level of TNFα was reduced in ozone-exposed MyD88−/− mice when compared with ozone-exposed wild-type mice, but not elevated in ozone-exposed TIRAP−/− mice when compared with FA-exposed TIRAP−/− mice. These observations support that TLR4-MyD88-TIRAP signaling pathway partially contributed to ozone-induced enhanced levels of pro-inflammatory cytokines/chemokines. Interestingly, when making comparison between ozone-exposed strains, KC, IL-1β and IL-6 levels in BALF from TIRAP−/− mice were significantly lower than that from either TLR4−/− or MyD88−/− mice; and BALF cytokine levels from MyD88−/− mice were lower than levels from TLR4−/− mice. The observed differences between strains suggest the existence of TLR4-independent signaling pathways that are dependent on these adaptor proteins. In addition, total protein levels were determined in BALF from all experimental animals as a marker of lung injury or permeability. Ozone exposure significantly increased protein levels in BALF from wild-type, TLR4−/−, MyD88−/− and TIRAP−/− mice when compared to air exposed animals. We did not observe significant differences in total protein levels between strains with this low dose of acute exposure to ozone (Figure 4). In conclusion, our observations support that TLR4−/−, MyD88−/− and TIRAP−/− contribute to the generation of pro-inflammatory factors within the airspace after inhalation of ozone.

### Inhalation of ozone increased the level of low molecular weight HA in the lung

**Figure 5:**
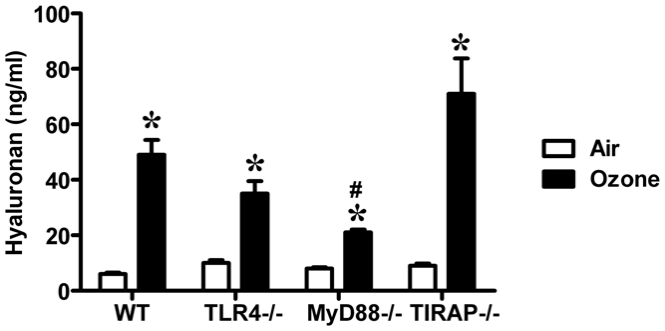
Level of HA in the BALF was increased after exposure to ozone. HA levels were significantly increased in all ozone-exposed groups (*p<0.01, vs. FA exposed group; #p<0.05, vs. O3-exposed WT, group, N = 4–5 per group).

Previously, we reported that 2 ppm of ozone exposure for 3 hours significantly increased HA level in BALF [20], [23] in a manner independent of intact TLR4. Consistent with the previous observation, 1 ppm of ozone exposure for 3 hours also increased HA levels in BALF in wild-type mice 24-hours after exposure. Similar elevated levels of HA were detected in BALF from ozone-exposed wild-type, TLR4−/−, MyD88−/−, and TIRAP−/− mice, when compared to air exposed mice. The levels of HA in BALF from ozone-exposed TLR4−/− and TIRAP−/− mice were not different from the levels of HA in BALF from ozone-exposed wild-type mice. Surprisingly, ozone–exposed MyD88−/− mice generated a lower level of HA in BALF than ozone-exposed wild-type, TLR4−/−, and TIRAP−/− mice. Baseline HA levels were not different among all air exposed strains (Figure 5). These results demonstrate that inhalation of ozone results in elevated levels of soluble HA and suggest that the level of HA in the BALF after ozone may be partially dependent on MyD88.

### HA- induced airway hyperresponsiveness is dependent on TLR4, MyD88 and TIRAP

**Figure 6:**
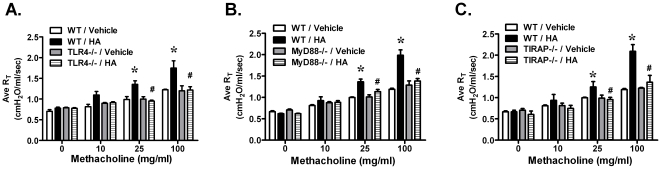
Airway reactivity to HA challenge was dependant on TLR4, MyD88, and TIRAP. HA increased AHR in WT mice, but not in (**A**) TLR4−/−mice, (**B**) MyD88−/−mice, or (**C**) TIRAP−/− mice (*p<0.05 vs. vehicle-WT; #p<0.05 vs. HA-WT, N = 5 per group).

Previous reports support that hyaluronan can function as an endogenous ligand of TLR4 in the context of non-infectious tissue injury [21], [34]. Our previous work demonstrates that TLR4 is required for both the response to ozone [11] and the response to short fragments of HA [23]. To determine whether the TLR4 adaptor proteins MyD88 and TIRAP contribute to the response to short fragments of HA, we directly challenged animals and measured methacholine sensitivity of the airways. As previously described, wild-type, TLR4−/−, MyD88−/−, and TIRAP−/− mice were challenged to either fragments of endotoxin-free HA or vehicle control by oropharyngeal aspiration. Two hours after exposure, the animals were characterized for AHR. We observed that all strains had similar levels of airway responsiveness to vehicle treatment. As previously reported, HA-treated wild-type mice demonstrate elevated AHR when compared to vehicle. However, TLR4−/−, MyD88−/− and TIRAP−/− mice were protected from the effects of HA challenge (Figure 6A, 6B and 6C). Airway sensitivity to methacholine challenge was not different among TLR4−/−, MyD88−/− and TIRAP−/− strains. These findings demonstrate that TLR4, MyD88, and TIRAP contribute to the biological response to short fragments of hyaluronan and contribute to the development of AHR.

### HA induced production of pro-inflammatory cytokines is partially dependent on TLR4, MyD88 and TIRAP

**Figure 7:**
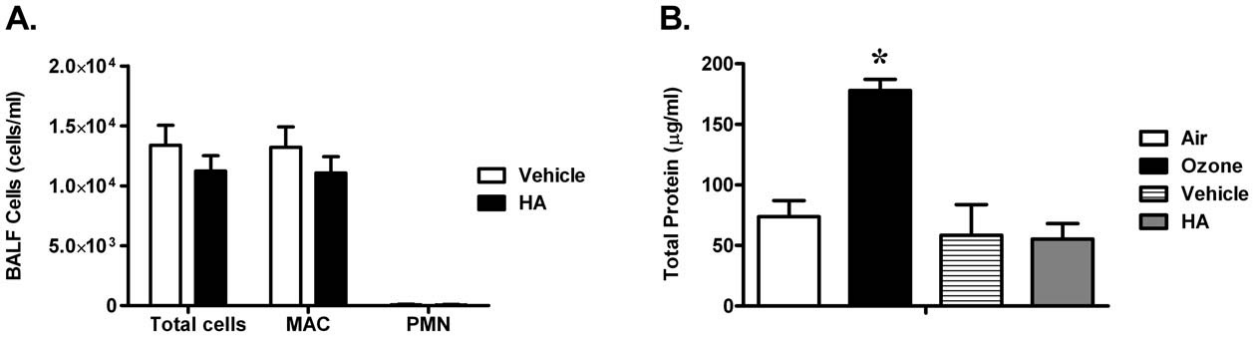
HA challenge was not sufficient for either neutrophilic inflammation or epithelial injury. (**A**) There were no observed differences in cellular inflammation in the airspace 2 hours after direct challenge to HA, (**B**) When compared to ozone challenge, HA challenge had no observed effect on the level of BALF total protein (*p<0.05, vs. FA exposed group, N = 5).

**Figure 8:**
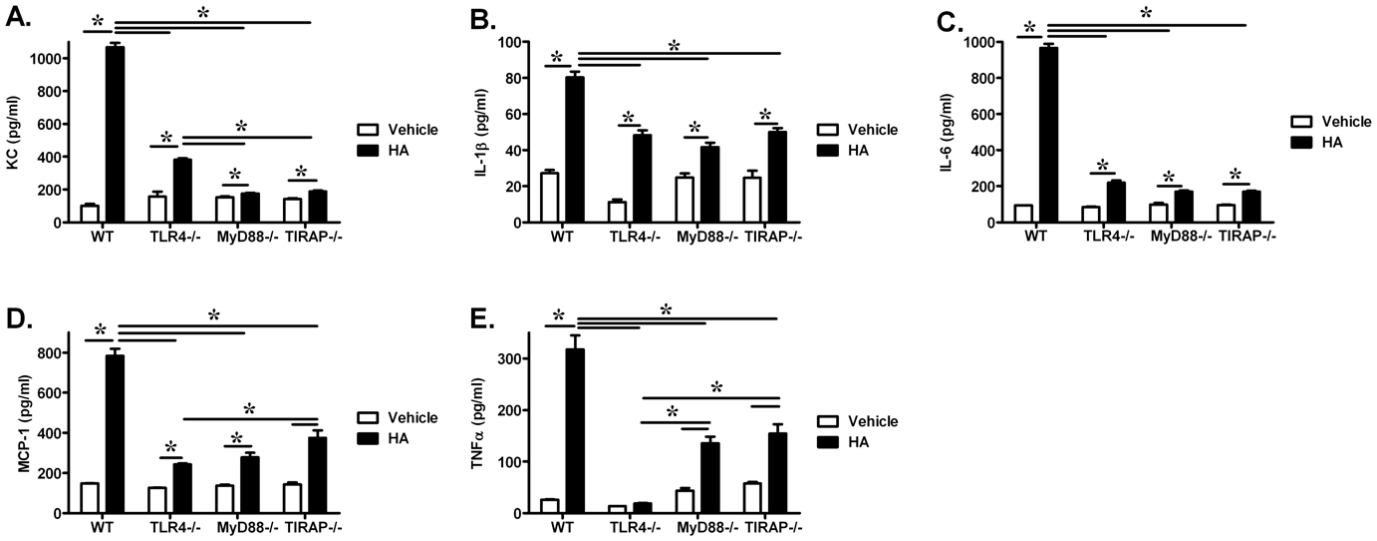
Direct challenge to hyaluronan fragments increased the level of pro-inflammatory factors in alveolar lavage fluid in a manner partially dependent on TLR4, MyD88, and TIRAP. The level of (**A**) KC, (**B**) IL-1β, (**C**) IL-6, (**D**) MCP-1 and (**E**) TNFα in BALF from vehicle or HA-challenged WT, TLR4−/−, MyD88−/− and TIRAP−/− mice were measured by luminex beads (*p<0.05, vs. FA exposed group; #: p<0.05, vs. O_3_-exposed group comparisons between strains; N = 4 per group).

Ozone exposure was associated with elevated neutrophils, total protein, pro-inflammatory cytokines/chemokines, hyaluronan, in addition to the development of AHR. Direct challenge to fragments of hyaluronan did result in development of AHR in wild-type animals. However, with exposure to hyaluronan fragments, we did not observe elevated levels of either total protein or neutrophils in the BALF (Figure 7A and 7B). After challenge to HA, we did observe elevated levels of pro-inflammatory factors, including KC, IL-1β, IL-6, MCP-1 and TNFα in the BALF of wild-type mice, when compared to vehicle challenge. Similar to the profile of cytokines after inhalation of ozone, the response to HA challenge was partially dependent on TLR4−/−, MyD88−/− and TIRAP−/−. The levels of these cytokines/chemokines in BALF were not different between HA-treated MyD88−/− and TIRAP−/− mice (Figure 8A, 8B, 8C, 8D and 8E). These observations support a central role of TLR4-MyD88-TIRAP in hyaluronan-induced production of pro-inflammatory cytokines in the BALF.

## Discussion

In this report, we demonstrate that the response to both ambient ozone and short fragments of hyaluronan are dependent on the TLR4-MyD88-TIRAP signal transduction pathway. We previously reported that the response to ozone is dependent of fragments of hyaluronan [20] and hyaluronan interaction with TLR4 [23]. We build on previous observations by demonstrating that the cytoplasmic adaptor molecules MyD88 and TIRAP are required for ozone-induced AHR. Specifically, we identify that TLR4-MyD88-TIRAP signal transduction pathway is a key determinant for AHR after either ozone or the direct delivery of hyaluronan to airspace. Additionally, we observe a similar profile of pro-inflammatory cytokines/chemokines dependent on TLR4-MyD88-TIRAP after challenge to either ozone or hyaluronan. Taken together, these observations support a striking role for HA-TLR4-MyD88-TIRAP signaling as a central transduction pathway in the development of reactive airways disease.

Our observations in the context of previous work provide additional support that HA, TLR4, MyD88, and TIRAP each contribute to the biological response to ozone and reactive airways disease. However, the dependence of each of these factors is not identical, which suggests that additional factors may contribute to the response to ozone. For example, TLR4-deficient animals are only partially protected from the effects of either ozone or hyaluronan when compared to MyD88-deficient animals. Thus, it appears that additional surface receptors participate to provide a complete innate immune response to ozone and may signal through conserved MyD88-dependent signaling. We and others have previously demonstrated that CD44 is the dominant receptor for hyaluronan and that CD44-TLR4 co-localize in context of non-infectious tissue injury [23], [34]. The functional role of the CD44-TLR4 surface complex remains poorly understood. Additionally, toll-like receptor 2 (TLR2) has been previously reported to contribute to both macrophage recognition of hyaluronan (21) and functional response to ozone [14]. After inhalation of 3 ppm ozone, TLR2 partially contributes to AHR, but not neutrophilic inflammation [14]. Characterization of the functional role of additional TLR-surface receptors in divergent models of inhaled oxidative stress could provide a more complete understanding of the role of innate immunity in lung injury. In addition, we observed that the level of TNF-α was significantly lower in the TLR4-deficient mice in response to hyaluronan when compared to wildtype, MyD88−/− and TIRAP−/− mice. This observation suggests the presence of a MyD88-TIRAP independent pathway, which may contribute to the inflammatory response to hyaluronan fragments. We speculate that additional TLR4 adaptor proteins including TIR-domain-containing adapter-inducing interferon-β (TRIF) or tumor necrosis factor receptor-associated factor (TRAF) may modulate the response to ambient ozone. However, the specific role of additional adaptor proteins and additional TLR surface receptors in response to ozone will be the focus of continued investigations.

Given the dominant effects of MyD88-TIRAP in this phenotype when compared to TLR4, consideration has to be given to the potential that additional ligand-receptor interactions may contribute to the response to ozone. For example, it is well recognized that IL-1 signaling utilizes the intracellular adaptor MyD88 [35], [36] and that the biological response to ozone is dependent on IL-1 [37], [38]. It is plausible that the dominant role of MyD88-TIRAP signaling and cellular response results from both ozone-induced generation of hyaluronan and IL-1. In this paradigm, hyaluronan would function as an endogenous ligand of TLR4 leading to MyD88-dependent signal transduction and additional MyD88-dependent signaling would arise from IL-1 receptor ligation. Thus, each ligand (HA and IL-1) individually may be sufficient to induce cellular responses causing AHR to develop. With respect to this scenario, depletion of both hyaluronan and IL-1 may be required to more effectively ablate ozone-induced airway hyperresponsiveness. Clear understanding of interdependence among TLR receptors, and the specific molecular ligands and cytoplasmic adaptor molecules antecedent to upregulation of signal transduction pathways could provide insight into development of novel therapies to limit over-zealous innate immune responses to microbial and environmental exposures.

There are fundamental differences between the two models that we have characterized for airway hyperresponsiveness. With respect to ozone, the animals were challenged to an environmentally relevant concentration level and response was phenotyped 24-hours after challenge. The delay in phenotyping is designed to model observations from studies of human exposure to ambient ozone, as previous epidemiological studies in humans demonstrate a lag of 24–72 hours after high levels of ambient ozone to the development of AHR [39]. In our animal model, we observe the development of ozone-induced AHR at 24-hours after exposure to ozone. After ozone, we do observe increased levels of hyaluronan in the lung even when delayed and investigated at a 24-hour time point. However, the response to hyaluronan is much more immediate [20]. Our previous work supports that ozone induces fragmentation of hyaluronan through oxidant stress [27]. We therefore speculate that ozone results in increased oxidant stress and fragmentation of hyaluronan and release into the airspace and into the sub-epithelial space. These fragments are thus positioned to increase production of pro-inflammatory cytokines/chemokines and recruitment of neutrophils. While we did not observe hyaluronan-induced recruitment of neutrophils into the airspace, we consider that this may be, in part, due to the timing of HA-induced neutrophilia. Interestingly, we observed the development of AHR in the absence of neutrophils in the airspace. While neutrophils are present in the airways after ozone exposure, the role of neutrophils in ozone-induced AHR remains somewhat unclear [40], [41]. The specific mechanism of hyaluronan fragments-induced airway hyperresponsiveness remains unknown, but we identify a central role of TLR4-MyD88-TIRAP.

The role of genes of innate immunity in context of non-infectious lung injury appears highly dependent on both the intensity and duration of stimulus and underlying host factors. High levels of ozone (3 ppm×2 hr) or prolonged exposure to low level ozone (0.3×72 hr) can result in considerable lung injury, which is associated with induction of mRNA expression of TLR4 [14], [42]. We do not observe induction of TLR4 mRNA with exposure to 1 ppm×3 hours ozone. It is not particularly surprising that the response to various models of lung injury is dependent on divergent host genetics. We recognize that the role of innate immunity in homeostatic maintenance of lung function is dependent on duration and intensity of injury. For example, low-level TLR4 signaling appears protective in some forms of oxidative lung injury [43], [44], [45], moderate TLR4 signaling facilitates the clearance of pathogens [46], [47], and excessive prolonged TLR4 signaling can augment lung injury [48]. Furthermore, in this study, we utilize a low dose exposure to hyaluronan fragments (25 ug/mouse or estimated 1 mg/kg), which activates genes of innate immunity and can induce AHR. Zhao *et al*. provide evidence that a much higher dose of hyaluronan (65 mg/kg) can result in neutrophil recruitment into the lung and induce lung injury. Interestingly, with high dose hyaluronan, TLR4-deficient mice had an enhanced response to hyaluronan suggesting both a TLR4-independent pro-inflammatory signaling pathway and that TLR4-signaling can contribute to induction of a negative regulatory pathway and induction of neutrophil apoptosis [49], [50]. Additional differences exist between these experimental protocols including the source and size of hyaluronan and timing of phenotyping. Clear understanding of the signaling pathways that contribute to both the induction and resolution of lung inflammation may provide insight into novel therapeutic approaches to lung injury.

Together, our results further highlight the important role of genes of innate immunity in recognition of the damage-associated molecular pattern hyaluronan. Hyaluronan is critical to the development of ozone-induced airway hyperresponsiveness and production of pro-inflammatory cytokines/chemokines. Understanding the mechanisms that regulate the response to ozone has important implications to public health. Recent evidence suggest that given anticipated alterations in the global climate, both the level and biological health impact of ambient ozone will be increased [6]. Furthermore, our mouse model provides fundamental insight into the role of damage-associated molecular patterns in reactive airways disease and novel potential targets for the treatment of asthma. In conclusion, our results support that the HA-TLR4-MyD88-TIRAP signaling axis contributes to the pathogenesis of reactive airways disease.
